# Self‐Care in Addiction Recovery: A Scoping Review

**DOI:** 10.1111/inm.70124

**Published:** 2025-09-04

**Authors:** Aina Folgueiras‐Vila, Maria‐Antonia Martorell‐Poveda, Maria del Señor Sesmilo‐Martínez, Pilar Vidal‐Massot, Laura Ortega‐Sanz

**Affiliations:** ^1^ Departament d'Infermeria, Grup d'Investigació en Infermeria Avançada (Caring) Universitat Rovira i Virgili Tarragona Catalonia Spain; ^2^ Servei d'Addiccions i Salut Mental de Reus, Hospital Universitari Sant Joan de Reus Reus Catalonia Spain; ^3^ Departament d'Infermeria, Hospital Universitari Institut Pere Mata, Institut d'Investigació Sanitària Pere Virgili (IISPV), CIBERSAM Universitat Rovira i Virgili Tarragona Catalonia Spain

**Keywords:** drug misuse, mental health recovery, rehabilitation nursing, self care, substance‐related disorders

## Abstract

Substance use disorders are a major threat to the health and quality of life of individuals. Self‐care is a strategy that affects health promotion and can be relevant in relapse prevention, as well as contribute to recovery in substance use disorders. This study aims to explore the diverse meanings of self‐care and the interventions that aligned with self‐care approaches for individuals recovering from substance use disorders. A scoping review was conducted following the Joanna Briggs Institute and PRISMA‐ScR guidelines. Studies published between 2014 and 2023 that explored self‐care (practices, definitions, or interventions) in adults (+18) undergoing treatment for substance use disorders, regardless of co‐occurring mental health conditions and across diverse cultural, geographical, and healthcare contexts, using various study designs were searched in CINHAL, Web of Science, Scopus, Psicodoc, PsycINFO, Cochrane Library, and reference lists, using MeSH terms “Self Care” and “Substance‐Related Disorders”, and free terms “addictions”, “addiction treatment”, “self care concept”, “interventions”, and “treatment”. Thirty articles were included in the final review. The meanings of self‐care are related to physical, behavioural, interpersonal, psychological and social levels. The variety of approaches and the integration of strategies such as mindfulness and cognitive‐behavioural group therapy highlight the importance of self‐care in substance use disorders. Self‐care is a key part of the recovery process and should be tailored to each person's unique experiences and needs. There is a need for consistent support in the implementation of self‐care strategies, in addition to creating interventions that address the complex lives of the specific populations.

## 1 Introduction

Substance use disorders (SUDs) pose a major global public health challenge, significantly affecting the well‐being of individuals, placing a burden on health systems worldwide (Delegación del Gobierno para el Plan Nacional sobre Drogas 2017). Beyond the individual, SUDs have repercussions for families and communities, leading to dependency, disability, and chronic health problems, as well as social consequences that transcend the user and affect their family and social environment (European Union Drugs Agency (EUDA) 2022; Organización Panamericana de la Salud (PAHO) 2023). These disorders pose a challenge for health systems, which must integrate early interventions, family support, and prevention strategies, which are especially relevant aspects for nurses and health professionals due to their key role in detection, accompaniment, and a comprehensive approach (Pedreira Crespo et al. 2011; European Union Drugs Agency (EUDA) 2022; Organización Panamericana de la Salud (PAHO) 2023).

According to DSM 5‐TR, an SUD is defined as a problematic pattern of substance use that results in clinically significant or functional impairment or distress (American Psychiatric Association 2022). Treatment options for SUDs encompass both clinical interventions and community‐based support. Clinical care includes psychological and pharmacological approaches, while community support involves mutual aid groups, therapeutic communities, and peer support programmes (Sampietro et al. 2023). Aligning with SUDs and their treatment, we find the complex concept of recovery, often misunderstood as cure. Recovery involves improvements in different areas such as symptomatology and functionality across social, occupational, and educational areas, sustained for at least 2 years (Ortega Sanz 2020; Uriarte Uriarte and Vallespí Cantabrana 2017). In addition, the concept of recovery extends beyond symptom reduction and abstinence, encompassing improvements in quality of life, mental and physical health, and social reintegration.

According to the Substance Abuse and Mental Health Services Administration (2012), recovery is a process of change whereby individuals improve their health and wellness, live a self‐directed life, and strive to reach their full potential. The World Drug Report 2024 points to a global increase in substance use, affecting more than 64 million people with SUDs. However, only a small portion receives treatment, with a greater disadvantage for women (UNODC 2024). Approximately 20% of countries worldwide have no screening programmes or brief interventions, and treatment coverage remains low and unequal. In this context, reaching the targets proposed by the WHO, such as reducing harmful drinking by 20% by 2030, seems difficult to achieve. It highlights the importance of integrating treatment into universal health coverage, increasing professional training, and ensuring accessible and evidence‐based interventions to address the health and social impact of SUDs (World Health Organisation 2024).

Taking these aspects into account, self‐care can be established as a key component in recovery from SUDs, especially when access to services is limited and people have to adapt to adverse contexts (Rhodes et al. 2015). Recovery from SUDs involves active decision‐making based on personal awareness and control, making it an essential tool for the day‐to‐day management of persistent health problems (Martínez et al. 2021); for example, meditation, therapy, or exercise not only strengthen physical and emotional well‐being, but also sustain the recovery process in the long term (Hill 2017).

Several authors have studied self‐care, providing depth and different perspectives to its meaning. Dean (1989) defines it as the behaviours that individuals undertake to promote or restore health, either independently or with professional guidance. It is characterised by autonomy and informed decision‐making, focusing on personal behaviours rather than broader environmental factors. The concept has evolved to emphasise active participation in health management, particularly for chronic conditions. This evolution highlights the critical role of individual actions in health protection and maintenance, especially for conditions requiring ongoing self‐management efforts (Dean 1989).

Another relevant approach is the one proposed from a nursing perspective by Dorothea Orem in her Self‐Care Deficit Theory. It is a comprehensive nursing theory where self‐care is understood as the actions or activities that are carried out and are oriented towards well‐being (Orem 1993). In this model, humans are conceptualised as rational beings capable of self‐reflection and symbolic thinking, emphasising the importance of self‐care in maintaining health and well‐being (Navarro Peña and Castro Salas 2010).

A more current contribution to self‐care is presented by González‐Vazquez et al. (2018), which contemplates a broader, multidimensional definition where self‐care is proposed as a pattern of relationship with oneself, the world, and others. This approach is the one we take as a reference to define self‐care and the interventions that would be included in it, such as mindfulness. According to this model, self‐care is understood as a multifaceted concept that includes three primary dimensions: external material factors, intrapsychic self‐care, and relational dynamics. It involves seeking positive experiences, maintaining a realistic self‐view, and fostering positive interactions with others (González‐Vazquez et al. 2018).

While self‐care has been extensively discussed and established as a mainstay in the management of long‐term health conditions, its specific role in recovery from addiction remains insufficiently addressed in the scientific literature (Nuño‐Solinis et al. 2013). A preliminary search in the MEDLINE (Ovid), JBI Evidence Synthesis, Cochrane Database of Systematic Reviews, and PROSPERO databases revealed a gap in the literature. While systematic reviews have addressed self‐care in the context of diabetes, cardiovascular diseases, and general mental health, there is a lack of comprehensive reviews that specifically examine self‐care interventions for individuals recovering from SUDs.

SUDs pose a significant public health problem with individual, social, and health effects. Although the concept of recovery has evolved towards a comprehensive and sustained vision over time, access to treatment remains limited. In this context, self‐care becomes relevant as a complementary tool to formal resources, with the capacity to adapt to different individual contexts. Despite its exploration in the general health care setting, its role in the recovery from SUDs has been under‐explored. This justifies the need for a review to identify and analyse the various definitions of self‐care and examine interventions that align with self‐care approaches for individuals recovering from SUDs through a scoping review methodology.

## 2 Aims

The overall objective of the review is to identify the different meanings of self‐care and the interventions that align with self‐care approaches for individuals in recovery from a SUD.

To address this aim, our secondary objectives are to:

– Describe the interventions and programmes intended for promoting and/or maintaining self‐care for individuals with a SUD.
– Explore the meanings of recovery as referenced in the literature, particularly from the perspective of and approach to SUDs.

## 3 Methods

The scoping review was chosen as the most appropriate methodology for this study as it allows extensive mapping of the existing literature on self‐care in people recovering from SUDs, an area that remains underexplored. Unlike systematic reviews, which focus on evaluating the effectiveness of specific interventions, scoping reviews are particularly useful for identifying knowledge gaps, clarifying key concepts, and mapping the range of interventions and definitions related to self‐care in the context of SUDs. This methodological approach aligns with the objectives of the study and ensures a comprehensive, yet structured, synthesis of the available evidence. The review was conducted following the recommendations established by the Preferred Reporting Items for Systematic Reviews and Meta‐analysis Protocols‐extension for Scoping Reviews (PRISMA‐ScR) (Tricco et al. 2018) and the methodological guidelines proposed by the Joana Briggs Institute (JBI) (Peters MDJ et al. 2024) based on the work by Arksey and O'Malley (2005) and extended by Levac et al. (2010).

The scoping review protocol was registered in the Open Science Framework on December 22, 2023, and updated on January 07, 2025; it is available for consultation: https://osf.io/8bcnx/.

### 3.1 Inclusion Criteria

Inclusion criteria were established using the PCC (Participants, Concept, Context) framework (Peters MDJ et al. 2024). Eligible documents met the following conditions:

### 3.2 Participants

Adults aged ≥ 18 years engaged in recovery through any form of treatment for SUDs. The coexistence of a mental health disorder is not considered an exclusion criterion in this review.

### 3.3 Concept

Documents that explore self‐care practices, definitions, and interventions related to SUDs.

### 3.4 Context

This review considers a wide range of documents on the meanings, practices, and interventions for individuals with SUDs, conducted in any geographical, cultural, or healthcare setting.

### 3.5 Type of Sources

This scoping review included documents published between January 2014 and December 2023 and a wide range of designs to comprehensively explore self‐care in individuals recovering from SUDs. Table 1 sets out the different categories considered.

**TABLE 1:** Types of sources.

	TYPES
Primary research studies	– Quantitative studies: Randomised controlled trials (RCTs), non‐randomised controlled trials, cohort studies, cross‐sectional studies, case–control studies. – Qualitative studies: Phenomenology, grounded theory, ethnography, qualitative description, action research. – Mixed‐method studies combining quantitative and qualitative approaches.
Secondary research and reviews	Systematic reviews, meta‐analyses, scoping reviews, and narrative reviews related to self‐care in SUDs.
Grey literature (to mitigate publication bias and capture non‐indexed evidence)	Conference proceedings, technical reports, dissertations, and policy documents.
Theoretical and expert opinion papers	Conceptual frameworks, position papers, and expert consensus documents addressing self‐care strategies. These were included only if they provided novel perspectives relevant to addiction recovery.

### 3.6 Search Strategy

Following the methodological guidelines of the JBI (Peters MDJ et al. 2024), the scoping review was carried out in three stages:

#### 3.6.1 First Stage

An initial search in two databases was conducted (SCOPUS and PubMed with Full Text) using MeSH (Medical Subject Headings) descriptors in the following Boolean phrase: (“Self Care” AND “Substance‐Related Disorders”). This search was carried out to identify relevant terms, analysing the words used in the titles and the abstracts of the documents found. This helped to design the different Boolean phrases used in the searches conducted in the second phase. Finally, it was decided to use MeSH descriptors such as “Self Care” and “Substance‐Related Disorders”, and the free search terms “addictions”, “addiction treatment”, “self care concept”, “interventions”, “treatment” and “addiction treatment”.

We incorporated into the manuscript the terms suggested by Kelly (2004) and Werder et al. (2021) to reduce stigmatisation around SUDs. However, some terms classified by Medical Subject Headings (MeSH) and other terms widely used in the scientific literature such as “addictions” have been kept.

#### 3.6.2 Second Stage

A search was carried out in: CINHAL (EBSCO), Web of Science, Scopus, Psicodoc (EBSCO), PsycINFO (EBSCO), Cochrane Library without applying any text or language filter. These databases were selected due to their relevance in the field of health sciences, psychology, and addictions. Documents published from January 2014 to December 2023 were included, as the nature of the research question neither focused on recent evidence nor on historical context. Different Boolean phrases defined in the first stage were used as a result of a preliminary search. Table 2 shows search strategies carried out between September 2024 and October 2024.

**TABLE 2:** Studies obtained by search and electronic database.

Search	APA PsycINFO	CINHAL	CochraneLibrary	Psicodoc	Scopus	Web Of Sciene
(“self care” OR “self care concept”) AND (“substance‐related disorders” OR addictions OR “addiction treatment”)	224	208	84	6	491	102
“self care” AND (“substance‐related disorders” OR addictions)	224	209	84	6	478	101
“self care” AND (“substance‐related disorders” OR addictions) AND interventions	100	68	74	2	155	45
(“substance‐related disorders” OR addictions) AND “self care” AND (interventions OR treatment)	168	123	81	3	321	72
(“self care” OR autocuidado) AND (addictions OR adicciones) AND (interventions OR “tratamiento de adicciones”)	89	32	59	2	109	44

#### 3.6.3 Third Stage

An analysis of the reference list of all the documents included in the second stage was conducted, taking into account that other relevant documents were identified and included in this scoping review.

### 3.7 Selection of Studies

To import and organise the documents obtained in the search and to eliminate duplicates, we used the bibliographic management and citation program Mendeley Desktop version 2.67.0 (Elsevier, Amsterdam, Netherlands).

The review and selection process was carried out in two phases: a first phase conducted by two of the researchers, in which the titles and abstracts were read following the established eligibility criteria. In this first phase, the relevant documents were saved in different files.

**FIGURE 1:**
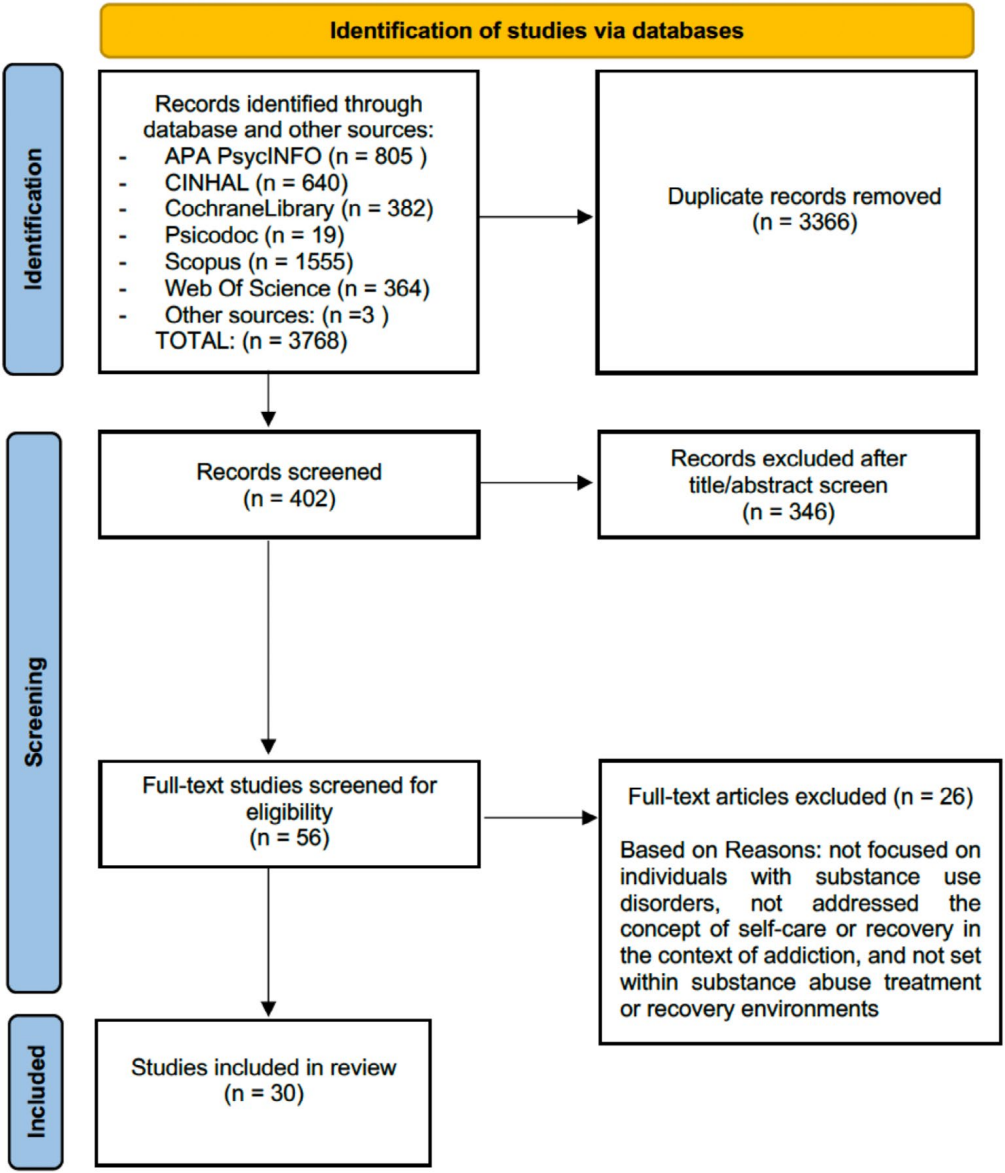
PRISMA flow chart for the scoping review (Page et al. 2021).

The second phase consisted of reviewing the full text of the documents selected previously and was carried out jointly by the researcher in charge of the first phase and a second reviewer to determine the documents for inclusion in the final review. Any discrepancies arising between the reviewers were resolved with the intervention of a third researcher, as well as re‐reading the full text and further discussion. When necessary, the authors of primary studies were contacted for further information or to clarify data, and even to obtain the full text once the document review process was completed. The complete results and the selection process are presented in a PRISMA flowchart (Figure 1) (Page et al. 2021).

Studies were excluded if findings included:

– High error rates or studies evaluating pharmacological treatments alone, without reference to self‐care strategies.
– Studies focusing only on child or adolescent populations.
– While the analysis of the quality of individual articles is not an objective of this review, documents with insufficient methodological transparency were discarded.

### 3.8 Data Extraction

Data extraction was conducted using a standardised tool (APPENDIX 1: Data Extraction Tool), adapted from the JBI framework (Peters MDJ et al. 2024). The data extracted was related to the main characteristics of the document, such as author/s, year of publication, country, study objective/s, methodology (design and participants), main results including details concerning the concept of self‐care, intervention, and treatment, as well as the tools used, and conclusions.

This process was carried out by two researchers in order to reduce potential bias in the final review; the discrepancies arising between these two researchers were resolved through discussion and with the help of the third reviewer to reach a consensus. Authors of papers were contacted to request missing or additional data when required.

## 4 Results

Thirty documents were included in the scoping review. The PRISMA diagram (Page et al. 2021) (Figure 1) shows the article selection process.

Table 3 provides an overview of characteristics, main results, and conclusions of the documents included in this review.

**TABLE 3:** Studies characteristics.

Reference and country	Main aim	Design and participants	Main findings	Conclusions
Bjelland et al. (2017) Norway	To explore the use of self‐help films in early addiction treatment, considering patient and therapist perspectives, as well as dyadic functions.	Qualitative exploratory research design. Patients (*n* = 12) and therapists (*n* = 22) in in−/outpatient addiction clinics.	It emphasises the connection between self‐care and mindfulness, suggesting that mindfulness training can enhance self‐care by improving individuals' understanding and management of their psychological states. The research implies that self‐care is an internal process that is crucial for managing thoughts, emotions, and behaviours in SUD treatment.	Use of a self‐help film may be a helpful adjunct to face‐to‐face therapy for patients who create a personally meaningful attachment to the film. Mindfulness/acceptance may offer a basic framework for such a connection to take place.
Brothers et al. (2023) UK/Canada	To understand the social‐structural determinants of injection‐related bacterial and fungal infections and identify opportunities for potential interventions.	Qualitative systematic review and thematic synthesis. Qualitative studies focusing on people who inject drugs (*n* = 26).	The review identified two main analytical themes: The first theme highlights macro‐environmental influences contributing to risk, while the second focuses on protective strategies employed by individuals, including mutual care and self‐care. Notably, while these self‐care strategies are protective, they can sometimes inadvertently introduce other health risks.	Injection‐related infections are largely influenced by modifiable social‐structural factors, including drug quality, criminalization, housing issues, limited harm reduction services, and problematic healthcare practices. Addressing these factors could significantly improve health outcomes for people who inject drugs.
Cardoso Leite Bica et al. (2019) Brazil	To understand crack users' perception of their health.	Descriptive‐exploratory research with a qualitative approach. Crack users registered in the Harm Reduction Strategy in Pelotas, Brazil (*n* = 14).	The main results highlight the subjectivity of health‐related issues and the importance of a comprehensive health care approach by health teams. The participants have a positive perception of their health, considering the use of crack as a way to alleviate the pain and suffering of everyday life.	There is a need for a more holistic and individualised approach to the care of crack users that takes into account their subjective perception of health and of their self‐care strategies.
Collins et al. (2022) Canada	To explore the impact of gendered occupational expectations on overdose risk among female and gender‐diverse drug users.	Case study. Women and gender diverse people who use drugs (*n* = 2).	Gendered work expectations, structural violence, and social contexts significantly influenced participants' experiences of overdose crisis. Participants faced challenges related to self‐care, work stress, and social expectations, which influenced their risk of overdose.	There is a need for gender‐specific interventions and harm reduction strategies that address the interrelated challenges faced by women and gender‐diverse people who use drugs.
Corser et al. (2022) Canada	To identify survival and wellness behaviours practiced by people who use methamphetamine and opiates simultaneously.	Qualitative approach, conducting semi‐structured individual interviews. Participants who use methamphetamine and opiates simultaneously (*n* = 22).	Participants made a number of considerations and adaptations to balance the perceived benefits and risks of their methamphetamine and opioid use through self‐care practices. Two general themes were identified: personal safety behaviours and interpersonal safety behaviours. Participants identified many gaps in the services available to meet their diverse needs.	Harm reduction and treatment responses must be robust and adaptable to respond to the diversity of substance use patterns among people who use methamphetamine and opiates simultaneously, so as not to perpetuate harm and not to be exclusive.
Dakin (2015) United States	The study examines the role of the intuitive function in addiction recovery, focusing on how intuition can shift the internal state dominated by addictive behaviours and aid in recovery.	Qualitative, intuitive, phenomenological methodology. Participants in recovery from alcoholism (*n* = 12)	Participants noted that synchronicities, dreams, and intuitive insights were pivotal in their decision to stop drinking. In long‐term recovery, intuition aids in problem‐solving, self‐care, and helping others, suggesting that cultivating intuition enhances self‐care behaviours by facilitating healthier choices and balanced impulse management.	The intuitive function plays a crucial role in addiction recovery, helping individuals recognise addictive behaviours and improve their relationship with the unconscious. Cultivating intuition is found to be effective in reducing impulsivity and enhancing decision‐making skills during the recovery process.
Ferentz (2015) United States	The main objective of the book is to provide clinicians with alternative treatment strategies for trauma survivors who exhibit self‐destructive behaviours.	The book is structured as a guide for clinicians, integrating theoretical insights with practical strategies. It includes cross‐references to a workbook that helps therapists apply these strategies in their practice.	It emphasises the importance of assessing and strengthening patients' self‐compassion to motivate change and healing. It also underscores the critical role of self‐care for therapists, defining it as a holistic approach to maintaining emotional and professional well‐being. Effective self‐care for therapists involves creating safety, managing personal triggers, setting appropriate boundaries, and practicing self‐compassion.	Fostering self‐compassion is crucial for patients dealing with self‐destructive behaviours such as SUDs. It also emphasises the importance of therapists maintaining self‐care to avoid vicarious traumatization.
González‐Baeza et al. (2023) Spain	To investigate associations between attachment, emotional regulation, childhood adversity, self‐care patterns and chemsex behaviour among GBMSM with drug‐related problems.	Cross‐sectional study. A group of GBMSM engaged in chemsex (*n* = 41) and a control group of GBMSM (*n* = 39).	Significant associations were found between insecure attachment styles, greater emotional dysregulation and a greater number of adverse life experiences with an increased likelihood of engaging in chemsex.	It highlights the importance of addressing attachment, emotional regulation and its mechanisms as self‐care, as well as childhood adversity in interventions targeting chemsex behaviour.
Gorvine et al. (2021) United States	This study aimed to examine the Whole Health resilience model in women recovering from SUDs, identifying potential interventions and research opportunities.	A qualitative research design with in‐depth interviews. Women in SUD recovery for two weeks or longer were conducted in nine different settings (*n* = 17).	Key findings included the identification of major themes such as social support, individual‐level cognitive and spiritual strategies, self‐care, stressors, priorities, self‐care needs and barriers, and trauma.	The development of integrated SUD interventions requires exploratory research to identify potential opportunities and barriers, as well as insight into the complex lives of women recovering from SUDs.
Keenan, Obekpa et al. (2023) Canada	To explore the experiences of women with OUD from first experiencing addiction to methadone treatment.	Qualitative, with interpretative phenomenological methodology. Women with OUD (*n* = 7).	Key nursing diagnoses include compromised adherence to treatment and anxiety. Nursing interventions focus on reinforcing therapeutic relationships, encouraging self‐care, and improving treatment adherence, highlighting the importance of self‐care in the recovery process for patients with SUDs.	Despite relapses, women showed remarkable resilience, demonstrating that self‐care, thoughtful decision‐making, and learning from past experiences are key to managing OUD.
Khantzian (2015) United States	To share insights and lessons learned from working with individuals who have experienced and recovered from addictive disorders.	A collection of clinical observations and personal reflections. The article presents case vignettes and anecdotes to illustrate key points.	Successful addiction recovery hinges on consistency and maintaining self‐care through basic health and safety habits. It emphasises that self‐care influences individual behaviour in risky situations and exists on a continuum across different populations.	Addiction recovery requires persistent engagement, self‐care, and a compassionate understanding of the complex genetic and environmental factors influencing mental health.
Kozasa et al. (2016) Brazil	To explore the potential of mindfulness practices in the promotion of health and in the treatment of physical and mental illnesses, particularly focusing on substance abuse.	Review chapter.	Mindfulness practices enhance metacognition and mental health, demonstrating neurological benefits through increased brain activity in attention‐related regions. Research shows effectiveness in treating mental health conditions and improving cognitive control, positioning mindfulness as a powerful self‐care strategy.	Mindfulness practices offer significant psychological benefits, showing promise in treating mental health conditions and potentially substance abuse. Regular meditation is associated with neuroplastic changes in attention and brain regions controlling impulse, suggesting that mindfulness could aid drug abuse treatment by enhancing sustained attention and impulse control.
Kraepelien et al. (2023) Sweden	To investigate the feasibility and preliminary effects of DPSC) for reducing alcohol consumption.	Feasibility study, prospectively registered as a clinical trial. Adults with problematic alcohol consumption (*n* = 36).	The DPSC approach aimed to increase treatment accessibility, maintain low clinician workload, ensure patient adherence, and potentially achieve effects comparable to therapist‐guided interventions.	DPSC for reducing alcohol consumption appears both feasible and preliminarily effective and should be further optimised and studied in larger trials
Melemis et al. (2023) Canada	To provide an understanding of the causes and stages of relapse, the role of CBT and mind–body relaxation in relapse prevention, and to introduce the five rules of recovery.	Book chapter	The study outlines relapse as a three‐stage process (emotional, mental, physical). It introduces the five rules of recovery as a framework for understanding and preventing relapse, highlighting self‐care as a crucial component encompassing emotional, psychological, and physical aspects, including addressing HALT conditions and using mind–body relaxation techniques.	Successful long‐term recovery hinges on understanding the relapse process, implementing life changes, seeking support, and utilising evidence‐based approaches like CBT and mindfulness. It emphasises the importance of early warning sign recognition, developing a support network, and adhering to the five rules of recovery for effective relapse prevention.
Meshesha et al. (2023) United States	To investigate the role of non‐substance activities in the treatment of alcohol use disorder (AUD) through patients' reported experiences.	A mixed (qualitative and quantitative) method: virtual focus group sessions and a self‐report survey. Participants with AUD (*n* = 21).	The most helpful activities were related to self‐care, social connections, acts of service, and creative outings. However, participants in group therapy reported less consistent support for activity engagement compared to those in individual therapy.	The participants believed that participation in substance‐free activities was an important component of their recovery, as well as to prevent relapse.
Murphy (2016) Middle East	To develop and evaluate a parenting and self‐care intervention for mothers living with HIV (MLH) based on findings from the Parents and children Coping Together (PACT) study.	Longitudinal study (PACT) followed by the development of an intervention (IMAGE: Improving Mothers' parenting Abilities, Growth, and Effectiveness).	It emphasises the importance of maternal self‐care, particularly managing depression and stress, as being crucial for effective parenting and maintaining positive family dynamics. Family routines, appropriate child responsibilities, parental monitoring and good communication are associated with better child outcomes.	Parenting interventions for MLH should prioritise establishing family routines, assigning age‐appropriate responsibilities, maintaining parental monitoring, and fostering positive communication. It emphasises that maternal self‐care, particularly managing depression and stress, is crucial for effective parenting and improving child outcomes.
Narenji et al. (2023) Iran	To design and determine the psychometric characteristics of a questionnaire to measure health‐promoting self‐care behaviours in patients recovered from drug addiction.	Cross‐sectional descriptive methodological investigation. Individuals who had recovered from drug addiction and were attending treatment clinics in western Mazandaran province (*n* = 245).	It defines self‐care as conscious and intentional activities to preserve life and improve health. It highlights its importance in drug addiction recovery, improving health, facilitating abstinence, and preventing relapses. It points out the influence of self‐care in multiple aspects of life, including physical, behavioural, intrapersonal, interpersonal, psychological and social areas.	The questionnaire developed has good validity and reliability and can be used to measure the self‐care behaviours of patients who have recovered from addiction, in order to provide appropriate solutions to prevent relapse to drug use.
Obekpa et al. (2023) United States	To describe recovery capital (RC) and health‐related quality of life (HRQOL) across five health dimensions among recovering residents taking medication for OUD and to identify predictors of these health dimensions.	Longitudinal study (Project HOMES). This specific analysis is cross‐sectional, examining data from a single time point. Participants from 14 recovery homes in Texas taking or willing to take medication for OUD (*n* = 358).	It emphasised the significance of self‐care in the recovery process for those with OUD, noting that self‐care challenges can negatively affect HRQOL. Improving self‐care capabilities is crucial for enhancing the overall well‐being of recovery home residents.	The study emphasises the need for attention to RC and HRQOL among individuals with OUD living in recovery homes, suggesting that enhancing these factors may improve recovery outcomes.
Pados et al. (2020) Hungary	The study aimed to explore the personal experiences and coping strategies of participants in the Dry November challenge.	Qualitative study using thematic analysis of diaries (*n* = 23).	The study identified three main themes: personal challenge, community support, and strategies for refusing alcohol. Self‐care emerged as a process of personal improvement and health consciousness, involving increased self‐awareness, health improvement, strategic social engagement, and community support.	The research demonstrated that during the Dry November challenge, participants employed strategies to manage social situations beyond simply refusing alcohol. The study highlighted the importance of community support and personal reflection in achieving temporary sobriety.
Peng et al. (2022) China	To examine the efficacy of CBGT in patients with alcohol dependence (AD).	Observational study. Male patients with AD who were randomly assigned to either the GCBT or the control group (*n* = 128).	The CBGT group exhibited significant improvements in insight and treatment attitude, chronic illness self‐awareness assessment scores, treatment adherence, and relapse rate compared to the control group.	CBGT is an effective approach for patients with AD, improving problem‐solving skills, self‐management and self‐care efficacy, positive responding and social function, leading to increased treatment adherence and a reduced relapse rate.
Phelps et al. (2018) United States	To explore the relationship between risk of SUD and self‐compassion, and to examine subscales of self‐compassion to determine their influence on this association.	Cross‐sectional study. (*n* = 477)	The low‐risk SUD group had a higher mean self‐compassion score than the high‐risk group. The study also identified strong correlations between SUD risk and all subscales of self‐compassion.	The risk of SUD is inversely associated with self‐compassion, suggesting that individuals with low self‐compassion may have an increased risk of SUD. Increasing self‐compassion may be a useful addition to SUD prevention and treatment interventions.
Price et al. (2019) United States	To examine the longitudinal effects of Mindful Awareness in Body‐oriented Therapy (MABT) as an adjunct to SUD treatment in women.	Randomised controlled trial. Women in intensive outpatient treatment for SUD at three community clinics (*n* = 187).	The study found that MABT, including self‐care activities in daily life, was associated with significant improvements in substance use, emotion regulation, craving, psychological distress, mindfulness, and interoceptive awareness skills.	MABT is effective in achieving longitudinal health outcomes and supporting women's recovery as an adjunct to community‐based treatment for SUD.
Raynor et al. (2017) Unites States	To explore perceptions of self‐care, recovery management, and preferred supports for mothers and fathers recovering from SUDs while actively parenting their children in the home environment.	Qualitative descriptive approach. Parents (*n* = 19; 11 fathers and 8 mothers) with at least one child between 6 and18 years old, and a minimum of 2 years in recovery from SUD.	The study finds different self‐care behaviours (SCB), identifying a range of strategies such as maintaining connections, physical health, spirituality, and emotional management. Participants described parenting as challenging, with varying levels of support.	SCB are critical for parents recovering from SUDs helping to maintain recovery and minimise past negative impacts on children. The study underscores the importance of these strategies while highlighting the need for further research into gender and minority‐specific variations in self‐care approaches.
Rodman (2016) United States	To document the substance abuse treatment experiences of transgender people.	Narrative study using individual interviews (*n* = 8).	Thirteen key themes were identified, ranging from making sense of addiction to programme policies. The research emphasises self‐care as a crucial, multidimensional approach to well‐being, encompassing emotional, physical, social, and spiritual aspects. It highlights that personalised self‐care strategies significantly contribute to resilience and support throughout the recovery process for transgender individuals facing unique challenges.	The study puts forward recommendations for substance abuse treatment programmes based on participant experiences and data analysis. These recommendations likely address the unique needs and challenges faced by transgender individuals in substance abuse treatment settings.
Romero‐Mendoza et al. (2022) Mexico	To describe the heroin and methadone use of intravenous heroin users of both sexes who have been in jail, to offer evidence for the formulation of health policy.	Ethnographic approach with semi‐structured open‐ended interviews. Heroin users (*n* = 33; 31 men, 2 women) from Michoacan, Oaxaca, and Mexico City who had been in prison.	Methadone use is interpreted as a critical self‐care mechanism to manage addiction, particularly when formal treatment is unavailable. It highlights how limited treatment access can lead to structural violence against users.	Compassionate methadone treatment and holistic attention should be considered as a way to meet patients' needs and mitigate their suffering, based on public health policy that allows for human rights‐based care.
Seabra et al. (2021) Portugal	To characterise the nursing care provided to a population dependent on psychoactive substances in a Specialised Technical Treatment Team.	Cross‐sectional, observational, analytical study with a quantitative approach. A population dependent on psychoactive substances, integrated in a pharmacological programme and in a nursing consultancy (*n* = 162).	Key nursing diagnoses include compromised adherence to treatment and anxiety. Nursing interventions focus on reinforcing therapeutic relationships, encouraging self‐care, and improving treatment adherence, highlighting the importance of self‐care in the recovery process for patients with substance use disorders.	The data shows that the diagnoses most addressed by nurses are therapeutic adherence, minimising consumption, and self‐care. Other domains may be under‐diagnosed in clinical practice according to the assessment of the consequences of substance use.
Sharif‐Brown (2023) United States	To develop a relapse prevention workbook curriculum that promotes a holistic approach to improve recovery outcomes for SUDs.	Dissertation. Development of a 12‐week group curriculum workbook.	Developed a 12‐week workbook curriculum for SUD recovery, focusing on mindset, self‐care, and spirituality. The self‐care module, comprising four lessons, emphasises a holistic approach including nutrition, physical exercise, and stress management.	A holistic approach to relapse prevention, incorporating mindset, self‐care, and spirituality, may enhance recovery outcomes for SUD. Further research is needed to evaluate the effectiveness of this approach.
Sierra Ortega et al. (2021) Spain	To generate an explanatory model for the onset of drug addiction from the principles of care.	Literature review and analysis of bibliographic sources in databases including Medline, Cochrane Database of Systematic Reviews, and Dialnet.	Drug use is framed as a form of self‐care, being a conscious and deliberate act. It defines self‐care as a continuous set of actions aimed at maintaining life and meeting needs, based on personal competence, and influenced by various factors.	It conceptualises drug use as a form of self‐care, emphasising the interplay between need, competence, basic conditioning factors, experience, and meaning in the development of addiction. It proposes using a care paradigm to understand drug addiction through the lens of self‐care and human characteristics.
Sinadinovic et al. (2014) Sweden	To compare an online personalised normative feedback intervention, eScreen.se, to a CBT‐based online extended self‐help intervention, Alkoholhjalpen.se, among individuals in the general population seeking help for problematic alcohol use.	Three‐armed parallel randomised controlled trial. Internet help seekers with at least hazardous alcohol use (*n* = 633).	All groups reduced alcohol consumption at the three‐month follow‐up, with effects remaining stable at six and 12 months. Per‐protocol analysis revealed that extended self‐help participants were more likely to move to lower alcohol use levels compared to brief intervention users and controls. These web‐based tools, particularly the extended self‐help intervention with CBT modules, can be considered forms of self‐care for managing alcohol consumption independently.	While intention‐to‐treat analysis showed similar effectiveness across all interventions in reducing problematic alcohol use, per‐protocol analysis indicated that CBT extended self‐help combined with other interventions was more effective than brief intervention or assessment alone in changing alcohol use patterns.
Swarbrick et al. (2023) Australia	To provide a framework for engaging young people in the co‐production of mental health services.	A comprehensive guide that outlines a co‐production approach. Intended for use by mental health service providers, policymakers, and young people themselves.	It emphasises the importance of recognising young people's expertise and ensuring inclusive collaboration. It also highlights self‐care as a personalised set of intentional activities crucial for maintaining physical, mental, and emotional well‐being, particularly in the context of youth mental health.	The guide underscores co‐production as a critical approach for developing youth‐centred mental health services, emphasising active youth involvement to create more relevant and impactful support.

The geographical distribution of the studies was as follows: United States (36.7%, *n* = 11), Canada (16.7%, *n* = 5), Sweden, Spain, and Brazil (6.7% each, *n* = 2). Other countries (26.5%, *n* = 8), including Australia, Mexico, Iran, China, Hungary, Norway, and Portugal. Regarding study design: Qualitative studies accounted for 40% (*n* = 12), including phenomenological and ethnographic approaches. Quantitative studies accounted for 33.3% (*n* = 10), predominantly cross‐sectional and randomised controlled trials (RCTs). Mixed‐method studies (3.3%, *n* = 1) provided an integrative perspective. Other sources (23.3%, *n* = 7) included literature reviews, book chapters, and one dissertation.

The populations studied were notably diverse, including individuals recovering from opioid use disorder (OUD) (Obekpa et al. 2023), people using methamphetamine and opioids simultaneously (Corser et al. 2022), women recovering from SUDs (Gorvine et al. 2021), and gay, bisexual, and other men who have sex with men (GBMSM) engaged in chemsex behaviours (González‐Baeza et al. 2023). Additionally, crack cocaine users in Brazil were included in the research (Cardoso Leite Bica et al. 2019), as well as alcohol‐dependent patients undergoing cognitive‐behavioural therapy (CBT) (Peng et al. 2022). Sample sizes ranged from small qualitative cohorts of fewer than 20 participants to larger cross‐sectional samples exceeding 200 participants, illustrating the breadth of research conducted across different contexts and populations.

The interventions examined in the studies varied widely, reflecting a range of strategies aimed at supporting recovery from SUDs. Cognitive‐behavioural group therapy (CBGT) was explored as an effective strategy for alcohol dependence recovery (Peng et al. 2022). Methadone treatment was highlighted as a critical self‐care mechanism for managing heroin addiction, particularly when formal treatment options were unavailable (Romero‐Mendoza et al. 2022). Recovery home programmes focused on improving health‐related quality of life for individuals recovering from OUD (Obekpa et al. 2023), while harm reduction strategies were addressed in the context of methamphetamine and opioid use (Corser et al. 2022).

Three themes were identified across the selected studies concerning the research questions of the scoping review (Table 4). Theme 1: Conceptualisations of self‐care in SUD recovery: includes the different significances found in the literature. Theme 2: Self‐care strategies and interventions: mentions of the different interventions or programmes related to promoting or maintaining self‐care. Theme 3: Meanings and trajectories of recovery: explains the different significances discovered in the review.

**TABLE 4:** Scoping review themes.

Author	Country	Self‐care meanings	Strategies and interventions	Recovery meanings
Bjelland et al. (2017)	Norway		X	
Brothers et al. (2023)	UK/Canada		X	
Cardoso Leite Bica et al. (2019)	Brazil		X	X
Collins et al. (2022)	Canada	X	X	
Corser et al. (2022)	Canada	X		
Dakin (2015)	USA			X
Ferentz (2015)	USA	X		X
González‐Baeza et al. (2023)	Spain	X		
Gorvine et al. (2021)	USA	X		
Keenan et al. (2023)	Canada	X		
Khantzian (2015)	USA	X		
Kozasa et al. (2016)	Brazil		X	
Kraepelien et al. (2023)	Sweden	X		
Melemis et al. (2023)	Canada	X	X	X
Meshesha et al. (2023)	USA		X	
Murphy (2016)	Middle East	X		
Narenji et al. (2023)	Iran	X		
Obekpa et al. (2023)	USA			X
Pados et al. (2020)	Hungary	X		
Peng et al. (2022)	China		X	
Phelps et al. (2018)	USA	X		
Price et al. (2019)	USA		X	
Raynor et al. (2017)	USA	X	X	
Rodman (2016)	USA	X	X	
Romero‐Mendoza et al. (2022)	Mexico	X	X	
Seabra et al. (2021)	Portugal			X
Sharif‐Brown (2023)	USA	X		X
Sierra Ortega et al. (2021)	USA	X		
Sinadinovic et al. (2014)	Sweden		X	
Swarbrick et al. (2023)	Australia	X		

### 4.1 Theme 1: Conceptualisations of Self‐Care in SUD Recovery

The first theme corresponds to the meanings of self‐care in the context of recovery (63.3% of studies, *n* = 19). The studies reviewed reveal a wide range of perspectives on self‐care in the context of SUDs, which can be categorised into three main trends: self‐care as a tool for emotional regulation and relapse prevention, self‐care as a relational and social process, and self‐care as an adaptive or maladaptive coping mechanism.

Several studies emphasise emotional regulation and relapse prevention, with Melemis et al. (2023) highlighting strategies to manage HALT (Hungry, Angry, Lonely, Tired) conditions and Kraepelien et al. (2023) introducing digital psychological self‐care (DPSC) to reduce alcohol consumption. Gorvine et al. (2021) focus on recognising triggers and managing stress, while González‐Baeza et al. (2023) link insecure attachment styles to risk behaviours in GBMSM engaged in chemsex, framing self‐care as an emotional regulation mechanism. Phelps et al. (2018) further explore self‐compassion as a protective factor against SUD risk.

Other studies highlight the relational and social dimensions of self‐care, such as the exploration by Rodman (2016) of the practices of transgender individuals emphasising emotional, physical, social, and spiritual resilience, and the discussion of Pados et al. (2020) concerning community support during temporary alcohol abstinence. Sharif‐Brown (2023) integrates self‐care into a group curriculum addressing nutrition, exercise, stress management, and spirituality, while Murphy (2016) focuses on maternal self‐care among mothers living with HIV to strengthen mental health and parent–child relationships. Swarbrick et al. (2023) advocate youth involvement in co‐producing mental health services to promote well‐being within supportive environments.

A third theme examines self‐care as an adaptive or maladaptive coping mechanism; Sierra Ortega et al. (2021) conceptualise it as a continuous set of actions for maintaining life but note that drug use can serve as a maladaptive form of self‐care when used to manage stress or distress. Romero‐Mendoza et al. (2022) highlight methadone treatment as an adaptive coping mechanism for heroin users facing structural barriers to formal care, while Collins et al. (2022) discuss how women and gender‐diverse individuals may use drugs for temporary relief from societal pressures, underscoring the need for gender‐specific harm reduction strategies. Khantzian (2015) defines basic health habits as critical elements of self‐care that influence behaviours in situations of risk, while Corser et al. (2022) frame efforts to regulate consumption during concurrent methamphetamine and opioid use as adaptive forms of self‐care. Finally, Narenji et al. (2023) emphasise conscious activities aimed at health preservation and relapse prevention during addiction recovery, showcasing how intentionality can transform maladaptive habits into healthier coping mechanisms.

### 4.2 Theme 2: Self‐Care Strategies and Interventions

In relation to the self‐care strategies and interventions identified in the reviewed studies (60%, *n* = 18), key approaches include mindfulness, CBT, and substance‐free activities, which support recovery by addressing psychological states, early relapse signs, and personal development. Additionally, drug use is sometimes viewed as a coping mechanism, offering temporary relief but posing health risks like HIV transmission.

CBGT‐based interventions emerged as one of the most studied, with evidence supporting their effectiveness in reducing relapse rates and strengthening self‐regulation skills in patients with alcohol dependence (Peng et al. 2022). Additionally, mindfulness‐based interventions, such as the Mindful Awareness in Body‐oriented Therapy (MABT) programme, have been linked to improvements in emotional regulation and a reduction in substance use (Price et al. 2019).

Another relevant approach identified in the studies is the use of digital tools for self‐care, such as online CBT modules, which allow individuals to manage their recovery autonomously. Research such as that of Sinadinovic et al. (2014) suggests that such interventions can be useful for the self‐regulation of alcohol use. In addition, harm reduction strategies were mentioned in several studies as key tools for self‐care, especially in high‐risk settings. For example, the study by Cardoso Leite Bica et al. (2019) found that some crack users perceive their use as a coping strategy to deal with suffering, highlighting the importance of a comprehensive and person‐centred approach.

### 4.3 Theme 3: Meanings and Trajectories of Recovery

The third theme identified in the literature corresponds to the meanings of recovery in SUDs, analysed in 23.3% of the studies reviewed (*n* = 7). Recovery is conceptualised not only as abstinence from substance use, but as a dynamic process involving emotional, mental, and physical dimensions (Melemis et al. 2023). In this regard, some research proposes holistic recovery models that include aspects such as nutrition, stress management, and spirituality (Sharif‐Brown 2023).

The relevance of recovery capital and its impact on the quality of life of people on medication for opioid use disorder is also highlighted (Obekpa et al. 2023). However, the literature also points to barriers to self‐care and recovery, such as a lack of access to adequate health services and the influence of social and occupational expectations on certain groups, such as women and gender‐diverse substance users (Collins et al. 2022).

The findings of this review show that self‐care in SUD recovery is a multidimensional concept, encompassing individual, relational, and structural strategies. While CBTs and mindfulness have been seen to offer benefits, there are emerging approaches, such as digital interventions and harm reduction, that require further research to assess their long‐term impact. Regarding the concept of recovery, the studies reviewed suggest that this should be understood beyond abstinence, integrating psychological, social, and environmental factors that influence the well‐being and autonomy of people with SUDs.

## 5 Discussion

The aim of this scoping review was to identify the variety of meanings of self‐care and interventions that align with self‐care approaches for recovering individuals with an SUD.

The review confirms that self‐care in SUD recovery is indeed a multidimensional concept encompassing intentional activities for physical, mental, and emotional well‐being (Swarbrick et al. 2023). It highlights the holistic nature of self‐care, integrating elements such as nutrition, exercise, and stress management (Keenan et al. 2023; Sharif‐Brown 2023), while also addressing social and community dimensions that foster supportive relationships (Pados et al. 2020; Raynor et al. 2017), and fundamental needs (Khantzian 2015; Melemis et al. 2023). Researchers emphasise the importance of mindfulness, emotional regulation, and self‐compassion as crucial components (González‐Baeza et al. 2023; Gorvine et al. 2021; Phelps et al. 2018). Notably, the literature acknowledges the resourcefulness of individuals with SUDs in managing their well‐being, even in challenging circumstances (Corser et al. 2022; Romero‐Mendoza et al. 2022), underscoring the adaptive potential of self‐care practices.

Building on this, we take as a reference the concept of González‐Vazquez et al. (2018), who consider self‐care as a pattern of relationship with oneself, the world, and others. This perspective not only aligns with the holistic nature of self‐care found in this review but also extends it by emphasising the balance between caring for oneself and for others. Early experiences of neglect or trauma are noted as potential barriers to healthy self‐care practices, which may manifest in maladaptive behaviours such as prioritising others' needs over one's own. This relational dimension resonates with the social aspects highlighted in the SUD recovery literature (Pados et al. 2020; Raynor et al. 2017), while the focus on intrapsychic self‐care aligns with the emphasis on mindfulness, emotional regulation, and self‐compassion (Gorvine et al. 2021; Phelps et al. 2018).

Key strategies for self‐care in SUD recovery include CBT and mindfulness‐based interventions. CBT, particularly in group settings, has shown promise in enhancing self‐care capacities and reducing relapse rates (Peng et al. 2022). Mindfulness‐based interventions demonstrate physiological and psychological benefits (Bjelland et al. 2017; Kozasa et al. 2016), with some researchers incorporating these activities into daily life through approaches like MABT (Price et al. 2019). These interventions support Orem's (1993) conceptualisation of self‐care as actions oriented towards well‐being. Melemis et al. (2023) offers a structured approach, integrating CBT and mindfulness for relapse prevention, emphasising their role in addressing maladaptive thought patterns and behaviours. These interventions align with the multidimensional framework of self‐care (González‐Vazquez et al. 2018), which includes external material factors, intrapsychic self‐care, and relational dynamics.

Digital tools have emerged as an innovative dimension of self‐care in SUD recovery. The role of technology is highlighted through DPSC (Kraepelien et al. 2023), with web‐based tools offering independent management of substance use through CBT modules (Sinadinovic et al. 2014). These digital interventions address barriers such as stigma and limited access to formal treatment, providing 24/7 support and enabling individuals to manage their recovery independently. These digital interventions align with the emphasis on autonomy and informed decision‐making in self‐care (Dean 1989).

Contrasting perspectives from additional studies reveal the importance of tailoring self‐care strategies to individual needs. Some researchers frame drug use itself as a maladaptive form of self‐care (Collins et al. 2022; Sierra Ortega et al. 2021), while others emphasise harm reduction approaches in high‐risk environments (Cardoso Leite Bica et al. 2019; Collins et al. 2022; Brothers et al. 2023), showing that the concept of recovery has evolved beyond mere symptom reduction and abstinence, encompassing improvements in quality of life, mental and physical health, and social reintegration (Uriarte Uriarte and Vallespí Cantabrana 2017). The incorporation of personalised strategies is crucial when addressing the needs of diverse populations (Collins et al. 2022; Rodman 2016). Factors influencing self‐care practices include need, competence, experience, and meaning (Sierra Ortega et al. 2021), as well as gendered work expectations and social contexts (Collins et al. 2022).

These diverse approaches highlight the multifaceted nature of self‐care in SUD treatment and recovery, underscoring the need for personalised and inclusive approaches that integrate physical, emotional, social, and spiritual dimensions.

## 6 Limitations

First, the heterogeneity of study designs and conceptualisations of self‐care rendered synthesis difficult. While scoping reviews do not usually assess the risk of bias, variability in study quality may have influenced our findings. In addition, publication bias cannot be ruled out, as studies with negative or non‐significant findings may be underrepresented.

To mitigate these limitations, we employed a rigorous search strategy and implemented a transparent selection process with inter‐rater agreement measures. Future research should consider complementary methodologies, such as meta‐analyses or systematic reviews, to further explore the effectiveness of self‐care interventions in SUD recovery.

## 7 Conclusions

The exploration of self‐care in SUDs reveals a multifaceted concept encompassing various meanings and interventions. Self‐care is broadly defined as intentional activities to promote physical, mental, and emotional well‐being, extending beyond abstinence to include holistic health practices. Interventions align with this comprehensive view, incorporating digital tools, mindfulness practices, harm reduction strategies, and personalised approaches. Web‐based CBT modules and mindfulness‐based interventions like MABT have shown promise in supporting recovery. The literature emphasises multidimensional strategies addressing the emotional, physical, social, and spiritual aspects of self‐care, particularly for diverse populations. Harm reduction approaches are recognised as being critical, especially in high‐risk environments. Structured approaches integrating CBT, mindfulness, and relapse prevention frameworks support abstinence and recovery. Pharmacological interventions, such as methadone use, are explored as forms of self‐care, emphasising individual agency. Engagement in substance‐free activities and CBGT have been identified as beneficial for recovery. The concept of recovery highlights the interconnectedness of various life domains in the recovery process. Overall, the literature underscores the need for personalised, comprehensive, and evidence‐based interventions that consider the diverse experiences and needs of individuals with SUDs in promoting and maintaining effective self‐care practices throughout their recovery journey.

Considering the results of the review and analysis, the areas that require further study and research are highlighted. Studies are required that can assess the long‐term impact of self‐care strategies on recovery. It would also be useful to develop theoretical models of self‐care in SUDs, as well as extend the evaluation of the effectiveness of digital and harm reduction interventions and incorporate interdisciplinary approaches. There is a need to develop studies that explore how differences in gender, sexual identity, and socio‐economic factors influence the effectiveness of self‐care interventions. The use of mixed methodologies that combine quantitative and qualitative data is required to better understand the phenomenon.

## 8 Relevance to Clinical Practice

This review emphasises the importance of integrating self‐care strategies into SUD recovery programmes, advocating for a shift from purely biomedical approaches to biopsychosocial and integrative models. Digital interventions offer potential for expanded access but require cultural adaptation and validation. The findings stress the need for personalised treatments considering individual differences, social factors, and structural barriers, particularly for women and gender‐diverse individuals who face unique challenges in recovery. This holistic approach aims to enhance emotional and social well‐being alongside traditional treatment methods.

This review emphasises the importance of integrating self‐care strategies into SUD recovery programmes, advocating for a shift from purely biomedical approaches to biopsychosocial and integrative models. In the context of nursing for mental health and addictions, the results provide a useful conceptual basis for incorporating self‐care as a component in treatment for people with SUDs. By recognising recovery as a multifactorial process, the role of nursing is reinforced not only in traditional treatment but also in the active promotion of psychosocial well‐being, patient autonomy, and care continuity. The findings stress the need for personalised treatments considering individual differences, social factors, and structural barriers.

## Author Contributions

All authors listed meet the authorship criteria according to the latest guidelines of the International Committee of Medical Journal Editors. All authors are in agreement with the manuscript. Authors' contribution to the article. AFV: designing the analysis, collecting data, performing the analysis, writing the manuscript, and reviewing process. MAMP: designing the analysis, collecting data, performing the analysis, writing the manuscript, and reviewing process. MSSM: reviewing process. PVM: reviewing process. LOS: designing the analysis, collecting data, performing the analysis, writing the manuscript, and reviewing process.

## Conflicts of Interest

The authors declare no conflicts of interest.

## Supporting information


Data S1.

APPENDIX 1.

## Data Availability

The data that support the findings of this study are available from the corresponding author upon reasonable request.
