# Supplementary material for: Self‐Care in Addiction Recovery: A Scoping Review
Source: Int J Ment Health Nurs. 2025 Sep 4;34(5):e70124. doi: 10.1111/inm.70124 (PMC12409770; doi:10.1111/inm.70124)
Supplement: Supplementary file 2 — APPENDIX 1. [file INM-34-0-s002.docx]

# **APPENDIX 1: Data Extraction Tool**

| SCOPING REVIEW  DETAILS |  |
| --- | --- |
| TITLE |  |
| AUTHOR AND YEAR |  |
| COUNTRY |  |
| OBJECTIVE |  |
| DESIGN |  |
| PARTICIPANTS |  |
| RESULTS  (CONCEPT OF SELF-CARE,  TOOLS,  INTERVENTION AND  TREATMENT) |  |
| CONCLUSIONS |  |
